# The Aim was About the Association with Psychiatric Disorders not on the Pathogenesis of Takotsubo - Author's Reply

**DOI:** 10.2174/1745017901915010005

**Published:** 2019-01-23

**Authors:** Federica Sancassiani, Mauro Giovanni Carta, Roberta Montisci, Sergio Machado, Maria Francesca Marchetti, Luigi Meloni

**Affiliations:** 1 Department of Medical Sciences and Public Health, University of Cagliari, Cagliari, Italy; 2 Laboratory of Panic and Respiration, Institute of Psychiatry of Federal University of Rio de Janeiro (IPUB/UFRJ), Rio de Janeiro, Brazil; 3 Physical Activity Neuroscience, Physical Activity Sciences Postgraduate Program - Salgado de Oliveira University, Niterói, Brazil

We would like to thank Dr. Finsterer and Dr. Stollberger for their commentary [1] on “Takotsubo Syndrome is Associated with Mood Disorders and Antidepressants Use, not with Anxiety and Impairment of Quality of Life due to the Psychiatric Disorder” [2]. We have found some of their suggestions really interesting, even their stimulating hypotheses rise some doubts about the legitimacy of some questions posed on our, admittedly, limited study. However, we believe it will allow us to define a few methodological knots.

The authors say “It is not a general consensus that Takotsubo (TTS) is more prevalent in patients with psychiatric disorders…”. As a matter of the fact, this is the focus of the study: our research (only) aims to bring useful news to verify such an association from a prevention and public health perspective. However, the importance of the results of the survey is confirmed and supported by the review of Nayeri [3] published a few months later of our work: the evidence agrees on an association between mood disorders and TTS. We hope that our and the concomitant results may have somewhat change the cited “…not general consensus…”. Obviously, if there had been a “general consensus”, the research would not have been conducted.

Discussing everything else, even intriguing, maybe too pretentious. Certainly, the evidence of a link between TTS and mood disorders may stimulate pathophysiolo- gical hypotheses and researches; *i.e.* about the weight of the vulnerability to the stress induced by a chronic psychiatric disorder and/or that of an acute psychiatric condition. Apparently, the Authors of the commentary have decided, we do not understand on the basis of which evidence, that only acute psychiatric conditions are important, and they have decided not to consider the literature on how chronic conditions can lead to stress vulnerability (even with excessive catecholamine resp- onse to specific triggers) [4, 5]. To address this hypothesis, we could stratify the sample by considering subjects with lifetime/point prevalence mood disorders, but it would decrease the power of the study. Thus, it would be impossible to verify this hypothesis by our sample. Nowise answering to this question was the goal of our study and this question is not so important from a public health perspective: the depressive disorders tend to recidivism, and people who have had an episode has a very strong probability of having others [6].

The authors are always concerned about the pathophysiological theories that were not in our objectives to verify, ask us about troponin. Consistent with the recruitment time, the Mayo Clinic diagnostic criteria we have adopted and correctly cited [7, 8] did not provide for the measurement of the troponin, which was included only in a subsequent set of Mayo Clinic criteria [9]. Of course, however, we have available data on troponin: all the TTS patients were admitted with suspected Acute Coronary Syndrome and all the biomarkers of myocardial necrosis were measured routinely. Nevertheless, as the study had no pathophysiological aims, we have not reported data on troponin in detail as well about all the cardiological parameters and biomarkers, nor about the frequency and duration of each psychiatric symptom used in the algorithm of the diagnosis of psychiatric disorders. A survey with the purpose to clarify the etiologic role of troponin would have reported the troponin data in (all the) cases and also planned an essay in (all the) controls. This clearly was not among the objectives of our study.

TTS is a fairly rare disease, so a case-control design with cases matched by sex and age with controls and adopting standardized criteria for diagnoses produces data that cannot claim to be conclusive (due to the sample size), but it allows reaching a stage that can be supported by meta-analyses or reviews on similar studies, as can indeed be seen in a recent review [3] precisely, because this is a standardized and easily replicable methodology. Although with recognized and well defined limits, this case-control model (by amplifying the power of the study thanks to the matching 4/1) makes it possible to point out a frequency of Major Depressive Disorder of 5/19 (26%) as worthy of interest because in the controls, similarly to the community [5], the frequency is only 6% (OR = 4.9, CI95% 1.02-23.5).

It is of interest also the data on the use of antidepressants, with a frequency of 3/19 (16%) in the cases compared to controls in which the frequency, quite similar to that in the community [10], is only 1% (OR = 14.1; CI95% 1.17-376.14). However, we have well honestly underlined that if one conducts an analysis standardizing for the co-occurrence of the diagnosis of Major Depressive Disorder, the difference is reduced and becomes not statistically significant. Even the odds ratio for antidepressant use is really higher than for Major Depressive Disorders, it does not allow us to affirm with certainty that the real association is the presence of the disorder and the antidepressant use is only a confounding factor. However, while we have stated in the conclusion that the study “...has confirmed the association of the Tako-Tsubo Syndrome with mood disorders and with depressive disorders in particular…”, in no way have we affirmed anything similar about the association with antidepressants, and we have clearly stated “...the results are not conclusive. The study suggests the need for studies with adequate sample size and methods to better define if there is an independent role of the two factors (depressive disorders and antidepressants use) and/or a possible interaction”. We have very clear in the mind that establishing an association is the first step but, according to the epidemiological methodology, establishing a causal link is quite another thing. For this reason, especially considering the prudence by which we have commented on the data, to read in the commentary that we “…conclude that antidepressants may be involved in the pathogenesis of TTS”, it leaves us somewhat amazed.

However, the above-cited review [3] reinforces the suspicion of an independent effect of antidepressants and suggests the urgency of researchers to clarify the role of antidepressants.

Finally, the authors have emphasized in their comme- ntary that psychiatric diagnoses are more than one per individual but this is really common in people with mental health problems [11], especially with mood and anxiety disorders [12]. Furthermore, in psychiatry, we have no etiological or anatomopathological criteria and the diagnoses are descriptive. Precisely for these reasons, the adoption of a standardized and reliable methodology, in a quite rare disease as TSS, allows more detailed answers on less frequent psychiatric diagnoses and/or about interaction with diagnoses and/or about the role of current or lifetime episodes, by increasing the power of the study due to the possibility to carry out further research and meta-analyses. We believe that other uncertain methodological paths can generate misunder- standings in a research world in which we see more and more often papers that do not define limited but clear objectives and do not consequently adopt standardized and reliable methods.
